# Multisite concordance of apparent diffusion coefficient measurements across the NCI Quantitative Imaging Network

**DOI:** 10.1117/1.JMI.5.1.011003

**Published:** 2017-10-10

**Authors:** David C. Newitt, Dariya Malyarenko, Thomas L. Chenevert, C. Chad Quarles, Laura Bell, Andriy Fedorov, Fiona Fennessy, Michael A. Jacobs, Meiyappan Solaiyappan, Stefanie Hectors, Bachir Taouli, Mark Muzi, Paul E. Kinahan, Kathleen M. Schmainda, Melissa A. Prah, Erin N. Taber, Christopher Kroenke, Wei Huang, Lori R. Arlinghaus, Thomas E. Yankeelov, Yue Cao, Madhava Aryal, Yi-Fen Yen, Jayashree Kalpathy-Cramer, Amita Shukla-Dave, Maggie Fung, Jiachao Liang, Michael Boss, Nola Hylton

**Affiliations:** a University of California San Francisco, Department of Radiology and Biomedical Imaging, San Francisco, California, United States; b University of Michigan, Department of Radiology, Ann Arbor, Michigan, United States; c Barrow Neurological Institute, Division of Imaging Research, Phoenix, Arizona, United States; d Harvard Medical School, Brigham and Women’s Hospital, Department of Radiology, Boston, Massachusetts, United States; e The Johns Hopkins School of Medicine, Russell H. Morgan Department of Radiology and Radiological Science and Sidney Kimmel Comprehensive Cancer Center, Baltimore, Maryland, United States; f Translational and Molecular Imaging Institute, Icahn School of Medicine at Mount Sinai, New York, United States; g University of Washington, Department of Radiology, Neurology, and Radiation Oncology, Seattle, Washington, United States; h Medical College of Wisconsin, Department of Radiology, Milwaukee, Wisconsin, United States; i Oregon Health and Science University, Advanced Imaging Research Center, Portland, Oregon, United States; j Vanderbilt University Medical Center, Vanderbilt University Institute of Imaging Science, Nashville, Tennessee, United States; k The University of Texas at Austin, Institute for Computational and Engineering Sciences, Department of Biomedical Engineering and Diagnostic Medicine, Austin, Texas, United States; l University of Michigan, Radiation Oncology, Radiology, and Biomedical Engineering, Ann Arbor, Michigan, United States; m Harvard Medical School, Massachusetts General Hospital, Athinoula A. Martinos Center for Biomedical Imaging, Department of Radiology, Charlestown, Massachusetts, United States; n Memorial Sloan-Kettering Cancer Center, Department of Medical Physics and Radiology, New York, New York, United States; o Memorial Sloan-Kettering Cancer Center, GE Healthcare, New York, New York, United States; p Hologic Inc., Sunnyvale, California, United States; q National Institute of Standards and Technology, Applied Physics Division, Boulder, Colorado, United States; r University of Colorado Boulder, Department of Physics, Boulder, Colorado, United States

**Keywords:** apparent diffusion coefficient, reproducibility, breast MRI

## Abstract

Diffusion weighted MRI has become ubiquitous in many areas of medicine, including cancer diagnosis and treatment response monitoring. Reproducibility of diffusion metrics is essential for their acceptance as quantitative biomarkers in these areas. We examined the variability in the apparent diffusion coefficient (ADC) obtained from both postprocessing software implementations utilized by the NCI Quantitative Imaging Network and online scan time-generated ADC maps. Phantom and *in vivo* breast studies were evaluated for two (${\mathrm{ADC}}_{2}$) and four (${\mathrm{ADC}}_{4}$) b-value diffusion metrics. Concordance of the majority of implementations was excellent for both phantom ADC measures and *in vivo* ${\mathrm{ADC}}_{2}$, with relative biases <0.1% (${\mathrm{ADC}}_{2}$) and <0.5% (phantom ${\mathrm{ADC}}_{4}$) but with higher deviations in ADC at the lowest phantom ADC values. *In vivo* ${\mathrm{ADC}}_{4}$ concordance was good, with typical biases of ±2% to 3% but higher for online maps. Multiple *b*-value ADC implementations were separated into two groups determined by the fitting algorithm. Intergroup mean ADC differences ranged from negligible for phantom data to 2.8% for ${\mathrm{ADC}}_{4}$ *in vivo* data. Some higher deviations were found for individual implementations and online parametric maps. Despite generally good concordance, implementation biases in ADC measures are sometimes significant and may be large enough to be of concern in multisite studies.

## 1 Introduction

The controlled sensitivity of nuclear magnetic resonance, and thus of MRI, to water diffusion provides medical researchers and clinicians a unique tool for measuring microscopic properties of tissue. In the realm of cancer in particular, quantitative diffusion-weighted MRI (DWI) is playing an ever-increasing role in both diagnosis and treatment response monitoring. In addition to providing information about tissue cellularity and microstructure, DWI has the advantages of not requiring the administration of an exogenous contrast agent and of requiring reasonably short acquisition times using standard echo-planar imaging techniques.

The simplest and most commonly used model for describing the MRI sensitive diffusion process is a monoexponential MRI signal decay as a function of the diffusion weighting (“b-value”) typically achieved with a pair of field gradient pulses as described by Stejskal and Tanner^1^ in 1965. This model assumes Gaussian diffusion behavior in isotropic tissue regions, characterized by an apparent diffusion coefficient (ADC) exponential decay constant. Despite the simplicity of this physical model, its practical implementation requires several choices that could affect the ADC measurements. These include masking of voxels for low signal-to-noise ratio (SNR) or poorness of fit; correction for nonideal imaging factors, such as low SNR effects, scanner nonlinearities, or diffusion weighting inaccuracies; and, for multi-b-value analysis, the choice of fitting algorithm may also be a source of variability.

For validation, reproduction of results, meta-analyses in multicenter studies, and consistency across multiple exams in longitudinal studies, it is essential that different analysis implementations (AIs) produce concordant results. Numerous studies have been published addressing repeatability and reproducibility of ADC measurements, mostly addressing the important aspects of acquisition repeatability^2^^,^^3^ and intra- and interreader reproducibility.^4^^,^^5^ For this work, the Image Analysis and Performance Metrics Working Group of the NCI Quantitative Imaging Network (QIN)^6^ undertook the ADC Mapping Collaborative Project (ADC-CP) to determine the effects of software platform and algorithm choices on ADC measurement through the analysis of common datasets by multiple institutions. The overall goal of the project is to quantify the cross-platform concordance of DWI parametric mapping software implementations. In this study, we present the results for ADC analyses performed on phantom and *in vivo* breast DWI, along with evaluation of the feasibility of centralized analysis of multicenter generated DWI parametric maps.

## 2 Materials and Methods

Overview: The ADC-CP was initiated and coordinated by the Breast Imaging Research Program (BIRP) at the University of California San Francisco (UCSF). Participants performed a prescribed set of DWI analyses on a common set of *in vivo* and phantom MRI datasets, generating derived parametric maps. These were submitted to the BIRP for centralized region-of-interest (ROI) and statistical analysis. Where available, parametric maps generated at scan time by on-scanner, manufacturer-provided software (“online” maps) were included in the central analysis.

### 2.1 Common DWI Datasets

Three groups of DWI datasets were analyzed in the ADC-CP: two b-value *in vivo* breast scans (Br2b), four b-value *in vivo* breast scans (Br4b), and four b-value phantom scans (Ph4b). Analysis metrics and MRI diffusion protocol details for all data are summarized in Table 1. All *in vivo* datasets were from the IRB approved American College of Radiology Imaging Network (ACRIN) 6698 trial^7^ and were used with the permission of ACRIN. *In vivo* image files were deidentified as per the requirements of the Health Insurance Portability and Accountability Act [Digital Imaging and Communication in Medicine (DICOM) standard, supplement 142], while preserving private metadata attributes necessary for DWI processing. DICOM images were curated and shared via the Cancer Imaging Archive.^8^ Each protocol group included scans from three MRI scanner manufacturers: Siemens Medical (SM), Philips Medical (PM), and General Electric Healthcare (GEHC). *In vivo* scans were multislice axial acquisitions with full biaxial breast coverage using standard two-dimensional (2-D) single-shot echo-planar imaging sequences. Group Br2b consisted of three studies: ID101 (GEHC, Signa HDxt, 3.0 T), ID102 (PM, Intera, 3.0 T), and ID103 (SM, Avanto, 1.5 T). Group Br4b consisted of four studies: ID201 (GEHC, Signa HDxt, 3.0 T), ID203 (GEHC, Signa HDxt, 1.5 T), ID205 (PM, Achieva, 1.5 T), and ID207 (SM, Avanto, 1.5 T). For all *in vivo* scans, a single b=0 image was acquired and non-0 b-value images were acquired with three orthogonal diffusion encoding directions. For all cases except ID203, standard on-scanner processing was used, resulting in trace images for each non-0 b-value and online generated ADC maps, and only the trace images were available for analysis. For ID203, the full set of directional DWI images was preserved, and no trace images or online ADC map were calculated.

**Table 1:** Dataset information for the ADC Mapping CP.

Group label	Description	N studies	b-values ($s/{\mathrm{mm}}^{2}$)	Scanner manufacturers^a^	Output parameters^b^	Analysis ROIs
Br2b	Two b-value, three direction bilateral axial breast	3	0, 800	GEHC, SM, PM	${\mathrm{ADC}}_{2}$	Multislice tumor
Br4b	Four b-value, three direction bilateral axial breast	4^c^	0, 100, 600, 800	GEHC, SM, PM	${\mathrm{ADC}}_{4}$	Multislice tumor
					${\mathrm{ADC}}_{\text{slow}}$	
					PerfFrac	
Ph4b	Four b-value, three direction diffusion phantom	3	0, 500, 900, 2000	GEHC, SM, PM	${\mathrm{ADC}}_{4}$	1-cm-diameter circles, 13 vials
					${\mathrm{ADC}}_{\text{hi-low}}$	

a Manufacturers: General Electric Healthcare (GEHC), Siemens Medical (SM), Philips Medical (PM).

b Output parameters: ${\mathrm{ADC}}_{\left\langle n\right\rangle }$: monoexponential ADC using all ⟨n⟩ b-values; ${\mathrm{ADC}}_{\text{hi-low}}$: monoexponential ADC using only highest and lowest b-values; ${\mathrm{ADC}}_{\text{slow}}$: monoexponential ADC using three highest b-values; and PerfFrac: fraction of b=0 signal attributed to fast-decaying perfusion component.

c An additional GEHC study with all directional DWI images but no trace images was included in Br4b.

The Ph4b datasets were of a diffusion phantom designed and constructed by the National Institute of Standards and Technology (NIST) and High Precision Devices (HPD Inc., Boulder, Colorado).^9^^,^^10^ This phantom consisted of an array of 13 20-mL vials in a spherical vessel filled with an ice–water mixture to maintain a controlled temperature of 0°C. Three vials were filled with water and ten vials were filled with solutions of the polymer polyvinylpyrrolidone (PVP) in deionized water,^11^ with two vials each at PVP mass fractions of 10%, 20%, 30%, 40%, and 50%. ADC values ranged from ∼1.1 to $0.12\times 10^{-3}\;{\mathrm{mm}}^{2}/s$. Scans were multislice coronal acquisitions at 3.0 T, using standard 2-D single-shot echo-planar imaging sequences. Diffusion encoding was applied on three orthogonal axes, with reconstruction of standard trace images at each b-value. Only the trace images were provided for analysis. Three datasets were provided: ID401 (GEHC, Discovery MR750, Memorial Sloan-Kettering Cancer Center, New York, New York), ID402 (SM, Trio, University of Colorado, Boulder, Colorado), and ID403 (PM, Ingenia, University of Michigan, Ann Arbor, Michigan). All phantom images used in this study were obtained by the DWI task force of the Quantitative Imagining Biomarker Alliance (QIBA) of the Radiological Society of North America (RSNA).

### 2.2 ADC-CP Participants

Participants in the ADC Mapping CP included 11 QIN sites and one non-QIN commercial group. A total of 15 DWI AIs were used (Table 2). Eight platforms were on-site developed private analysis packages programmed in MATLAB^®^ (The MathWorks Inc., Natick, Massachusetts; six implementations “AI-MAT1” to “AI-MAT6”), IDL^®^ (Exelis Visual Information Solutions, Boulder, Colorado; “AI-IDL”), or C++ (“AI-C++”). Five implementations utilized free, publicly available analysis packages: 3D Slicer DWModeling module of the SlicerProstate extension^19^ (Brigham and Womens Hospital; two implementations “AI-3DSl1” and “AI-3DSl2”), AFNI (University of California, San Diego, Analysis of Functional Neuro Images; “AI-AFNI”), OsiriX ADCMap plugin (Stanford; “AI-OsX1”), and QIBAPhan (RSNA/University of Michigan; “AI-QIBA”). Two implementations were commercially available analysis packages: Aegis™ (Hologic Inc., Sunnyvale, California; “AI-Aegis”) and OsiriX plugin IB Diffusion™ (Imaging Biometrics, Elm Grove, Wisconsin; “AI-OsX2”). Source websites for the publicly available software packages are included in the references in Table 2.

**Table 2:** DWI quantitative AIs included in the ADC Mapping CP.

AI ID	Data groups	Base language or platform	AI publicly available	Parametric map format	Multi-b fit (function)^b^
AI-IDL	All	IDL^®^	No	DICOM	NLS-GX (curvefit)
AI-MAT1	Br2b, Br4b	MATLAB^®^	No	MATLAB^®^	NLS-TRF (lsqcurvefit)
AI-MAT2	All	MATLAB^®^	No	MATLAB^®^	Log-linear
AI-3DSl1	All^a^	3D Slicer DWI Module^12^^,^^13^	Yes	DICOM	NLS-LM
AI-MAT3	Br2b, Br4b	MATLAB^®^	No	MATLAB^®^	Log-linear (lscov)
AI-QIBA	Ph4b	QibaPhan1.3^14^	Yes	DICOM	Log-linear (lscov)
AI-OsX1	All^a^	OsiriX-ADCMap^15^	Yes	DICOM	Log-linear
AI-MAT4	Br2b, Br4b	MATLAB^®^	No	ANALYZE	Log-linear
AI-CPP	All	C++	No	DICOM	Log-linear
AI-AFNI	Br2b, Ph4b	AFNI^16^	Yes	NIfTI	NA
AI-MAT5	Br4b, Ph4b	MATLAB^®^	No	NIfTI	Log-linear
AI-OsX2	All	OsiriX-IB Diffusion^17^	Yes	DICOM	Log-linear
AI-MAT6	All	MATLAB^®^	No	MATLAB^®^	Log-linear (polyfit)
AI-3DSl2	All^a^	3D Slicer DWI Module^12^^,^^13^	Yes	NRRD	NLS-LM
AI-Aegis	All^a^	Aegis (C++)^18^	Yes	DICOM	Log-linear

a No perfusion-fraction ($P_{f}$) analysis performed on Br4b.

b Multi-b fitting methods: NLS-GX=nonlinear least squares using gradient expansion, NLS-TRF=NLS using trust-region-reflective, NLS-LM=NLS using Levenberg–Marquardt, and log-linear=linear fit or regression of log(S). Base software package function name is given where known.

### 2.3 ADC-CP Parametric Maps

For the purpose of the ADC-CP, the basic monoexponential decay model for the MRI signal intensity from an isotropic tissue region was assumed

$$S(b)=S_{0}\times e^{-b\times \mathrm{ADC}},$$ (1)

where S(b) is the signal intensity at a diffusion weighting b, $S_{0}$ is the true signal for no diffusion weighting, and ADC is the apparent diffusion coefficient. For practical considerations, methods for the derivation of the estimated ADC from a DWI acquisition can be separated into two cases: two b-value analyses wherein the ADC is solved explicitly via the following equation:

ADC={log[S(b1)]−log[S(b2)]}/(b2−b1), (2)

and multi-b-value analyses where fitting of the data to Eq. (1) must be done to determine the ADC. The choice of algorithm for fitting multi-b-value data, as well as the choice of any masking parameters, was left to the participating sites.

Site analysis consisted of generating a set of parametric maps from pixel-by-pixel analysis of each DWI dataset. Analyses performed for each data group are listed in Table 1. For all cases, a monoexponential ADC map utilizing all images was computed: ${\mathrm{ADC}}_{2}$ for Br2b, and ${\mathrm{ADC}}_{4}$ for Br4b and Ph4b groups. In addition, for the Br4b group, a perfusion minimized analysis was performed.^20^ For this analysis, the three nonzero b-values were used to estimate the “slow” or tissue diffusion signal using Eq. (1) for $b\geq 100\;s/{\mathrm{mm}}^{2}$, giving $S_{0\text{slow}}$ and ${\mathrm{ADC}}_{\text{slow}}$ as the fitted parameters characterizing the slow signal decay. The fraction of the signal attributable to a fast-decaying perfusion component was then calculated as

$$P_{f}=[S(0)-S_{0\text{slow}}]/S(0),$$ (3)

and parametric maps were generated for ${\mathrm{ADC}}_{\text{slow}}$ and $P_{f}$. For the Ph4b group, a two b-value decay coefficient, ${\mathrm{ADC}}_{\text{hi-low}}$, was also calculated using only the b=0 and $2000\;s/{\mathrm{mm}}^{2}$ images.

In addition to the parametric maps provided by the analysis sites, scanner manufacturers’ software (“online”) ADC maps were evaluated when they were provided with the original DWI data. This included the ${\mathrm{ADC}}_{2}$ for the Br2b group, ${\mathrm{ADC}}_{4}$ for the Br4b datasets with trace images (three of four studies), and ${\mathrm{ADC}}_{4}$ for the Ph4b group.

### 2.4 Centralized ROI Analysis

All parametric maps were submitted to UCSF through a secure box system. No restrictions were placed on the choice of file format, and formats included DICOM (N=7), Neuroimaging Informatics Technology Initiative (NIfTI; N=2),^21^ Nearly Raw Raster Data (NRRD; N=1),^22^ Analyze (Mayo Clinic; N=1),^23^ and MATLAB^®^ (N=4). Prior to concordance analysis, all maps were converted to a UCSF in-house modified multiframe DICOM format allowing integer or floating point data, along with storage of an analysis mask. Slice order was detected automatically for file formats that do not include orientation information and was reversed if necessary to match the slice order of the source images. ADC scaling was detected automatically by comparison with a reference UCSF ADC map, and scaling factors were set in the metadata (DICOM rescale slope attribute) to produce ADC maps in common units of $10^{-6}\;{\mathrm{mm}}^{2}/s$. No manipulation of the actual map pixel data was done except for floating point formats (MATLAB^®^ implementations) in which pixels with a “not-a-number” value were reassigned to 0.0 and masked out for analysis.

**Fig. 1:**
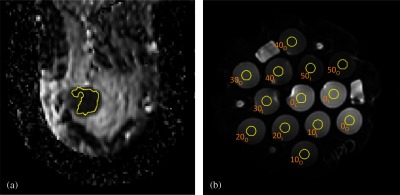
Typical ROI placements for (a) breast studies and (b) phantom studies, shown on ${\mathrm{ADC}}_{4}$ maps. (a) A single representative slice from a multislice breast tumor ROI. The ROIs were drawn referencing the high b-value DWI and an accompanying DCE subtraction image. Calculated mean ADC was taken over the full multislice ROI. Phantom ROIs shown in (b) are single-slice, 1-cm-diameter circles labeled with the PVP concentration (0% to 50%) and a position subscript: C=center, I=inner, and O=outer.

ROI analysis was performed using standardized ROIs across all parametric maps (Fig. 1). For the *in vivo* breast cancer scans, a multislice, whole-tumor region defined for use in the primary study was used. For the phantom scans, ROIs were defined on the middle slice of each scan using 1-cm-diameter circular regions on each of the 13 sample vials. ROIs were applied to the parametric maps yielding mean values of the diffusion metrics for each analysis platform. All centralized analysis was done using software developed by the UCSF lab in IDL^®^.

### 2.5 Statistical Analysis

For each metric, pairwise within-subject coefficient of variation (wCV) was calculated between all implementation pairs to establish groups of implementations with similar results (intragroup wCV<0.1% between all AI pairs). As no ground truth values could be established for the *in vivo* assessed DWI metrics, individual implementation concordance could only be evaluated from the percent difference of each ROI measurement from a consensus reference value for that measurement. This method was also used for the phantom scans even though reference ADC values were available, both for consistency of presentation and to avoid complications from scanner- and position-dependent ADC effects. A full analysis of the phantom ADC data relative to the ground truth reference values is presented by Malyarenko et al.^24^ Reference value calculation for each of the metrics is described in Sec. 3. The two-tailed student’s T-test was used to test for significant differences among different implementations.

## 3 Results

### 3.1 Practicalities

From the 12 participating institutions, monoexponential ADC maps for the Br2b and Br4b groups and perfusion minimized ${\mathrm{ADC}}_{\text{slow}}$ values for Br4b were provided for 13 analysis platforms. Nine platforms from eight institutions also provided perfusion-fraction maps for the Br4b group. The Ph4b data group was analyzed on 11 platforms, 10 generating both ${\mathrm{ADC}}_{4}$ and ${\mathrm{ADC}}_{\text{hi-low}}$ parametric maps while one provided only ${\mathrm{ADC}}_{4}$. All sites were able to process DICOM image sets from all three vendors, but interpretation of the no trace, full directional data (Br4b, ID203) was challenging for several sites due to unfamiliarity with this format. After specification of the image storage order for this case, all sites were able to program their implementations to process this data, though in some cases we noted discrepancies in the results as shown in Sec. 3.2.

### 3.2 Breast Scans

**Fig. 2:**
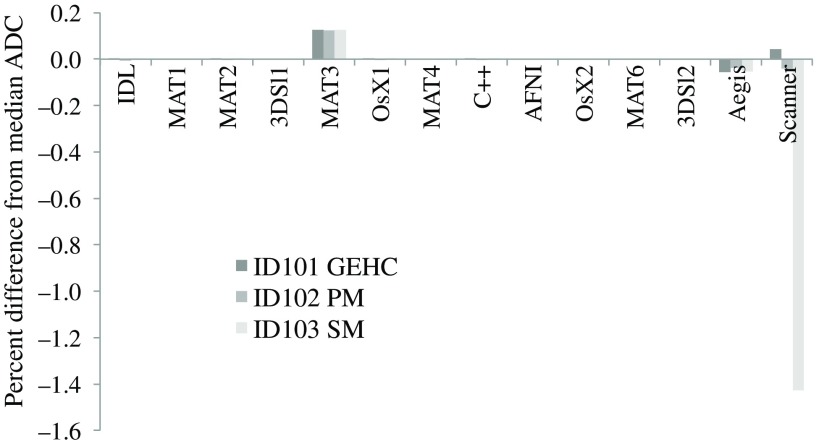
Concordance of two b-value *in vivo* ADC measurements across 13 offline AIs and online scanner-generated maps. Plotted is the percent difference for each ROI mean value from the median value for that measurement for all offline AI. Eleven offline AI had essentially identical results (wCV<0.003%) and thus show no offsets on the plot. The SM online ADC had a −1.4% bias relative to the consensus median value.

For the Br2b ${\mathrm{ADC}}_{2}$ metric, a majority of the AI (11 of 13) gave essentially identical results (maximum wCV<0.003%). For each dataset, the median ADC value from all offline results was used for the reference value for concordance. Figure 2 shows the percent difference from these reference values for each AI’s mean ROI ${\mathrm{ADC}}_{2}$ measure for each of the three Br2b scans. AI-MAT3 had a consistent 0.12% positive bias relative to the median, while AI-Aegis varied from −0.04% to −0.06%. The GEHC and PM online maps were within 0.05% of the respective median values, but the SM map had a −1.4% bias.

**Fig. 3:**
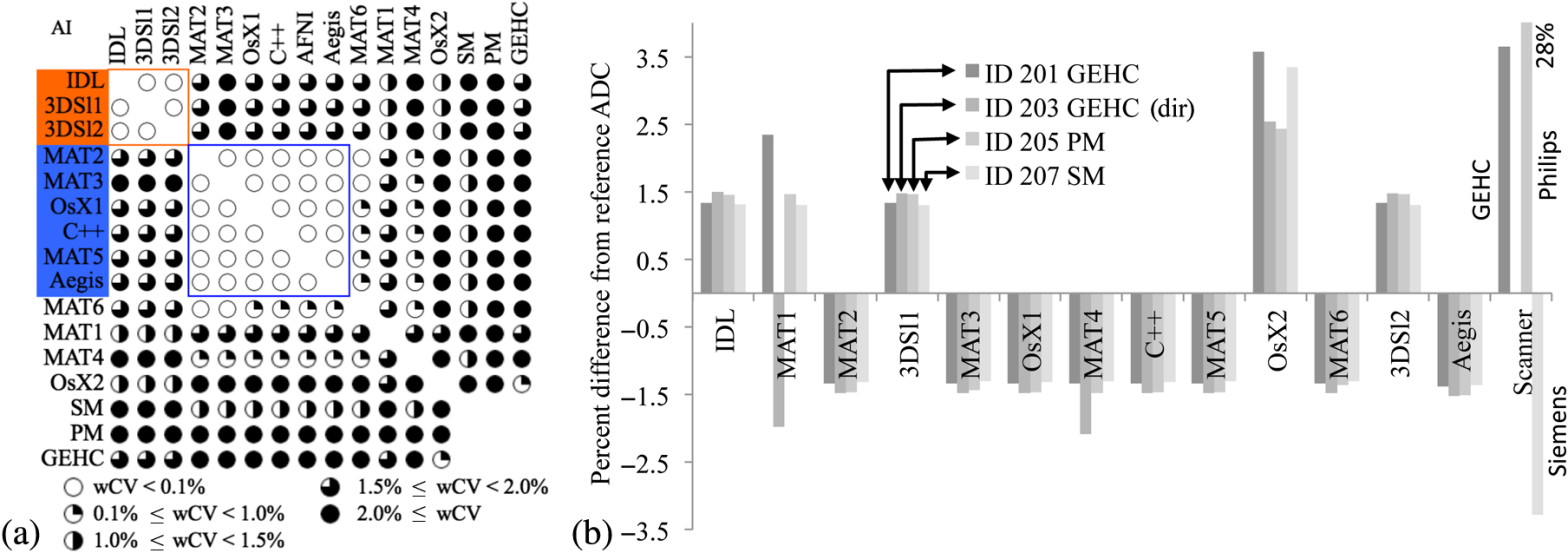
*In vivo* four b-value ${\mathrm{ADC}}_{4}$ ROI analysis results. (a) Pairwise wCV matrix for all implementations, shown graphically from wCV<0.1% (white circles) to wCV>2% (fully black circles), with groups A and B indicated. (b) Percent difference from the consensus ${\mathrm{ADC}}_{4}$ values for each of four datasets, for each implementation and online map. Mean difference in ADC between groups A and B was 2.8%. The 28% deviation on the PM online ADC was due to a DICOM header corruption problem.

More variations were observed among platforms in the Br4b analyses. Figure 3(a) shows graphically the pattern of agreement among platforms given by the pairwise wCV measures. A majority of implementations (9 of 13) fell into two groups when using a threshold of wCV<0.1% among all group members. Group A consisted of three AI (AI-IDL, AI-3DSl1, and AI-3DSl2) with wCV<0.01%, while group B consisted of six AI (AI-MAT2, AI-MAT3, AI-MAT5, AI-OsX1, AI-C++, and AI-Aegis) with wCV<0.1%. For each dataset, a reference value was calculated as the average of the mean value for group A and the mean value for group B. Figure 3(b) shows the percent difference from these reference values for ${\mathrm{ADC}}_{4}$ from each implementation for the Br4b datasets. ${\mathrm{ADC}}_{4}$ values differed significantly between groups A and B [2.8%±0.2% (mean±SD), p<0.003], and up to 5% between nongrouped sites. Two of the four nongrouped implementations, AI-MAT4 and AI-MAT6, had only small variations (wCV<0.13%) from the group B values, while AI-MAT1 and AI-OsX2 showed more variability both between scans from different vendors and from the reference values. Two implementations, AI-MAT1 and AI-MAT4, had slightly anomalous results for ID203 (GEHC), believed to be due to different handling of the full directional diffusion data. Scanner-generated ${\mathrm{ADC}}_{4}$ maps were available for the three datasets with trace images. GEHC and SM maps gave mean ROI ADC values of +3.6% and −3.3%, respectively, from the reference values, while the PM online map had a 28% offset. Further investigation revealed that this large deviation was due to loss of the DICOM rescale slope data employed by PM for parametric map intensity scaling. This loss appeared to have occurred during data transfer between the scanner and the imaging site’s PACS system.

**Fig. 4:**
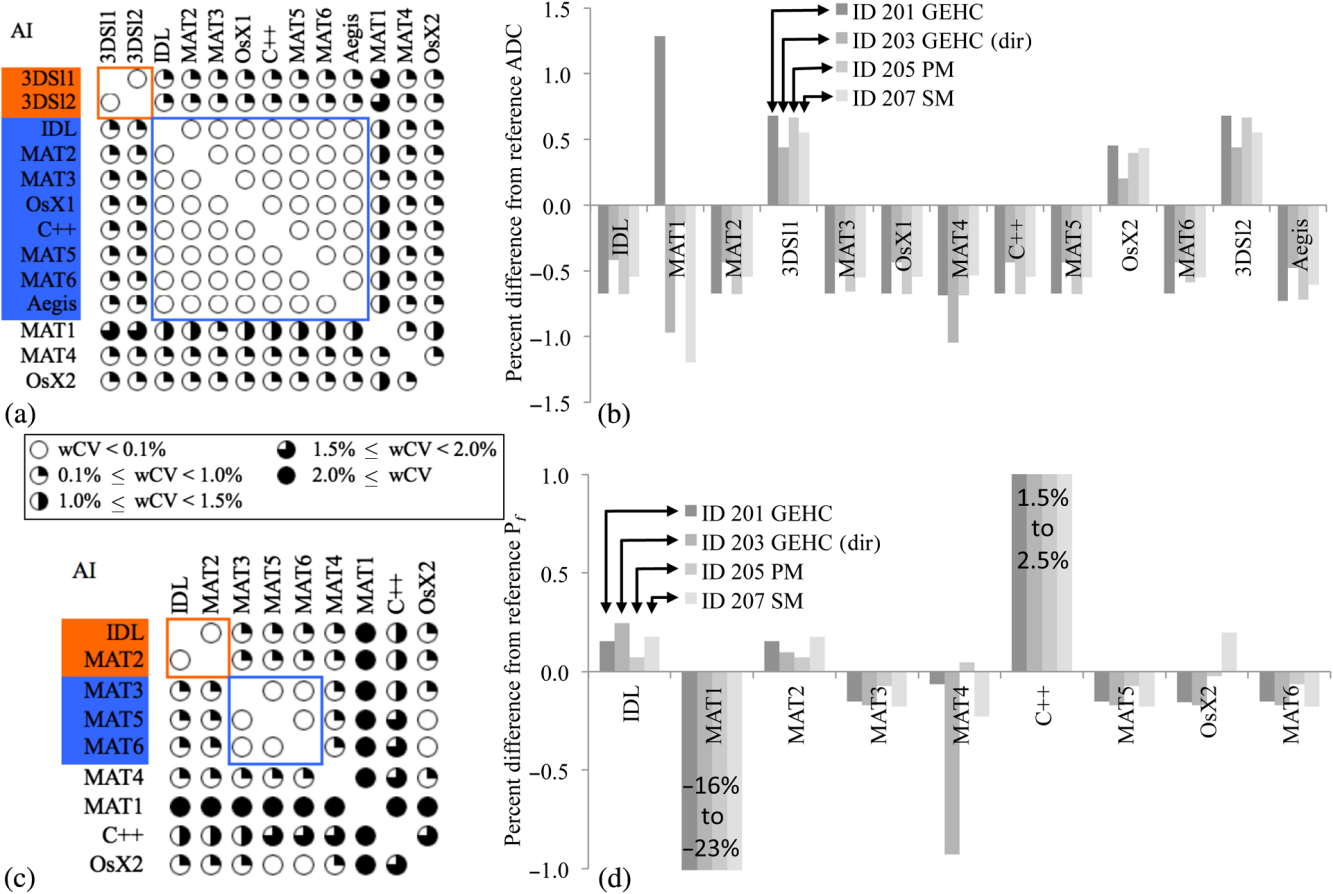
wCV and ROI mean concordance results for the Br4b data group perfusion minimized analysis. For ${\mathrm{ADC}}_{\text{slow}}$, (a) shows the pairwise wCV matrix with groups with wCV<0.1% indicated and (b) the corresponding data for differences in mean ROI ${\mathrm{ADC}}_{\text{slow}}$. Group results showed smaller variations than for ${\mathrm{ADC}}_{4}$. For $P_{f}$, (c, d) groups were less well defined, except for the three MATLAB^®^ AI indicated, which were nearly identical (wCV<0.01%). The small positive biases for AI-C++ were identified as due to use of a biexponential model.

Results for the perfusion minimized analysis tissue ADC (${\mathrm{ADC}}_{\text{slow}}$) were similar to the ${\mathrm{ADC}}_{4}$ results [Figs. 4(a) and 4(b)]. For wCV<0.1% grouping, AI-IDL switched from group A to B, and AI-MAT6 was also now included in group B. Overall differences were generally smaller than for ${\mathrm{ADC}}_{4}$ but still statistically significant: 1.2%±0.2% (mean±SD, p<0.003) difference between groups A and B and maximum individual differences of any implementation <±1.3% relative to the reference value. Perfusion fraction ($P_{f}$) was a nonstandard metric and was implemented on nine platforms. Two groups were again evident, though with different membership [Fig. 4(c)]: group A (wCV=0.04%) composed of AI-IDL and AI-MAT2 and group B (wCV<0.01%) with MATLAB^®^ implementations AI-MAT3, AI-MAT5, and AI-MAT6, with a small difference among the groups [0.29%±0.10% (mean±SD), p<0.03]. Figure 4(d) shows the concordance for the $P_{f}$ metric results. $P_{f}$ results from AI-MAT1 showed large deviations (−16% to −23%) from the consensus reference, indicating possible errors in the software implementation that was developed on-site for this CP. AI-C++ had a positive bias of 1.5% to 2.5%, which was found to be due to implementation of a biexponential decay model for this calculation. All other measures fell within ±0.25% of the reference values, except for the AI-MAT4 result for the GEHC directional diffusion dataset with a −0.9% deviation. No online parametric maps were available for the perfusion minimized analysis.

### 3.3 Phantom Scans

Analyses of the three Ph4b phantom datasets, ID401 (GEHC), ID402 (SM), and ID403 (PM), were submitted from 11 AI for the four b-value ${\mathrm{ADC}}_{4}$ metric and 10 AI for the two b-value ${\mathrm{ADC}}_{\text{hi-low}}$. For AI-C++, only the ID402 results were included, as a problem in the DICOM encoded ADC maps for ID401 and ID403 resulted in incorrect ROI ADC values in the centralized analysis. In a separate analysis completed after the encoding bug was fixed, these results were in concordance with the other implementations.^24^ Online maps for ${\mathrm{ADC}}_{4}$ were available for all three phantom datasets, but only ID403 (PM) included an online map for ${\mathrm{ADC}}_{\text{hi-low}}$. Results for the two b-value ${\mathrm{ADC}}_{\text{hi-low}}$ were practically identical across all implementations. The maximum pairwise wCV among postprocessing implementations using all 39 ROI measurements from the three datasets was 0.04%. Looking at the percent difference of each ROI measure from the nine site median values, AI-QIBA showed a similar clinically insignificant bias (0.05%) to that seen in the Br2b datasets for AI-MAT2. The results from the online PM ${\mathrm{ADC}}_{\text{hi-low}}$ map were very close to the offline reference median values, yet showed a consistent but clinically insignificant bias of $0.31\pm {0.02\times 10}^{-6}\;{\mathrm{mm}}^{2}/s$ across all ADC values. This gave a maximum percent difference of −0.25% for the lowest ADC sample.

**Fig. 5:**
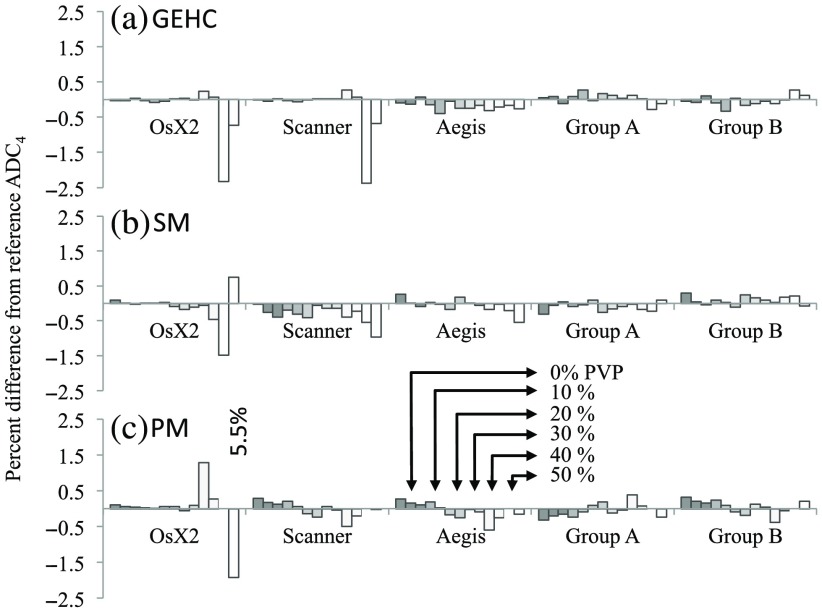
Percent difference from reference ${\mathrm{ADC}}_{4}$ for Ph4b measurements for (a) GEHC, (b) SM, and (c) PM scans. The reference value for ${\mathrm{ADC}}_{4}$ for each individual ROI is the average of the groups A and B mean ${\mathrm{ADC}}_{4}$ values for that ROI. ROIs are ordered from highest ADC (0% PVP) to lowest ADC (50% PVP), left to right, for each AI or group. Concordance is excellent, except for a few measurements on the lowest ADC vials ($\mathrm{ADC}<0.25\times 10^{-3}\;{\mathrm{mm}}^{2}/s$).

For the ${\mathrm{ADC}}_{4}$ measures, paired wCV measurements over all phantom measurements gave similar groups to the Br4b results. Differences within and between the two postprocessing implementation groups were smaller than for the breast scans. The maximum wCV was 0.04% for group A (AI-IDL, AI-Sl1, AI-Sl2, and AI-MAT6) and 0.01% for group B (AI-MAT2, Al-QIBA, Al-OsX1, and Al-MAT5), and the between-group root mean square percent difference in ADC values for all 13 ROIs was 0.29%, 0.30%, and 0.62% for GEHC, SM, and PM scans, respectively. There was no significant bias among the ROI mean ADC values from the two groups (p=0.15, 0.07, and 0.19 for GEHC, SM, and PM scans, respectively). Figure 5 shows the differences from reference ${\mathrm{ADC}}_{4}$ (average of the mean group A and mean group B results) for the three Ph4b datasets. While differences are in general very small (<0.5%), individual excursions were as high as 5.5%, with the highest differences on the lowest 2 ADC values ($\mathrm{ADC}<0.25\times 10^{-3}\;{\mathrm{mm}}^{2}/s$). Only the SM online map showed a statistically significant deviation from the reference values, with a small negative bias of −0.31%±0.25 (mean±SD, p=0.001). Only the PM dataset analysis showed a trend with ADC value in the difference among the analysis groups, with group A tending to underestimate ADC relative to group B for higher ADC values and over estimate at lower. A linear regression of the percent difference between the groups versus the mean ROI ADC gave a slope of 1% per $1.0\times 10^{-3}\;{\mathrm{mm}}^{2}/s$ with $R^{2}=0.35$.

## 4 Summary and Discussion

Overall, the QIN ADC Mapping Collaborative Project demonstrated good agreement between the majority of postprocessed (“offline”) and scanner-generated (“online”) ADC implementations, while revealing several sources of discrepancies among different platforms. With the exception of isolated outliers, mostly attributable to metadata errors rather than algorithmic differences, the largest discrepancies observed were between online and offline parametric maps. The most consistent bias was for Siemens scanner acquisitions, where the online maps gave ADC values lower than consensus reference values derived from the offline maps. These ranged from −0.3% (phantom 4b) to −1.4% (*in vivo* 2b) to −3.5% (*in vivo* 4b). Based on communication with Siemens, the most likely explanation is the use by the online ADC algorithm of detailed image sequence information to calculate a more accurate b-value than the nominal value stored in the DICOM metadata, which is used for all offline calculations. A higher true b-value, obtained by accounting for diffusion and imaging gradient cross terms, will result in a lower calculated ADC value, as we observed. The General Electric online maps for the *in vivo* four b-value ADC also showed a marked discrepancy from the consensus reference (+3.5%), though it agreed identically with one of the offline implementations (AI-OsX2, OsiriX IB Diffusion plugin).

The biases we report for the *in vivo* breast scans are of comparable magnitudes to measures of repeatability and reproducibility reported in breast ADC studies. Aliu et al.^2^ reported a wCV of 11% in a repeatability study on normal volunteers, while Spick et al.^5^ and Clauser et al.^25^ found wCV values between 5.0% and 8.5% for breast tumor ADC measurements. In the ACRIN 6698 trial, whole-tumor ADC test–retest repeatability was 4.8%.^3^ Our results indicate that choices in ADC analysis algorithm or between online and offline analysis platforms will have nonnegligible effects on breast ADC measures and should be considered in addition to biases arising from image acquisition when interpreting findings in breast DWI studies.

A consistent finding was a grouping of a majority of the implementations for multi-b ADC estimation into two groups with very similar results within-group but significant differences between the two groups. Based on the descriptions of the methods provided by each site, this appeared to be primarily driven by the choice between “log-linear” fitting, wherein a linear least-squares fit is done on the log of the image intensities, and a nonlinear least-squares fit of the untransformed data to the exponential diffusion equation. For the *in vivo* scans, the difference in implementations resulted in significant differences (p<0.003) of 2.8% for ${\mathrm{ADC}}_{4}$ and 1.2% for the ${\mathrm{ADC}}_{\text{slow}}$ in the perfusion minimized analysis. Our results are comparable to those reported by Zeilinger et al.^26^ using different methods. While the grouping based on pairwise wCV was also apparent in the four b-value phantom ADC_4_, no significant difference was found for the resulting ADC measures (p=0.22). We speculate that this may be due to the higher noise level and heterogeneity within each ROI in the *in vivo* scans giving a greater sensitivity to the fitting algorithm selection, but further work is needed to identify the cause. Finally, given the lack of ground truth values for the *in vivo* scans, it is important not to equate discrepancies with errors in the presented work, except in those cases where specific error sources could be identified. In particular, while the choice of reference values for most of our ROI result plots as the average of the two prominent AI groups allows easy visualization of the differences between the AIs, it also can lend an appearance of preference to those AIs over the “nongrouped” results.

The QIN ADC Mapping CP also highlighted some practical challenges of multicenter ADC analyses and centralized analysis of postprocessed parametric maps. For example, several sites had to implement code for the ADC-CP to analyze the less common full directional dataset, which may have resulted in somewhat higher variability in the results for those scans. While saving of directional data for DWI is not currently a common practice in clinical trials, it may become more so in the future given ongoing work on improving reproducibility of multiplatform DWI by gradient nonlinearity correction^27^^–^^29^ and distortion correction.^30^ Another lesson learned was the criticality of preservation of DICOM metadata for quantitative DWI. In particular, the case of lost scaling information in a Philips scanner-generated ADC map illustrates that significant errors can result from metadata corruption. While the nature of this project resulted in easy recognition of this problem, in a clinical trial setting, it might have gone unnoticed. Finally, the centralized analysis of parametric maps for this CP was greatly complicated by the multitude of file formats currently employed for the storage of these objects. Adoption of a common format, such as the parametric map DICOM object,^31^ would aid meta-analysis of ADC data obtained from multicenter studies. Use of DICOM, specifically for ADC map storage, was addressed in a companion cooperative project.^24^

A limitation of this study was the restriction to the monoexponential decay model, with the simple extension to a perfusion minimized ${\mathrm{ADC}}_{\text{slow}}/P_{f}$ calculation. For *in vivo* situations where the simple Gaussian diffusion model breaks down, several more complex models are currently employed such as biexponential models,^32^ including intravoxel incoherent motion,^33^^,^^34^ stretched exponentials,^35^ and kurtosis.^36^^,^^37^ As model complexity increases, dependency on AI choices will also increase. An additional limitation of this study stems from the choice of a single organ, the breast, for the *in vivo* datasets. As breast DWI is challenging, due largely to limitations in SNR, fat suppression quality, motion, and other artifacts, we consider these datasets a challenging test of the fitting algorithms’ robustness. However, the results presented are only indirectly relevant to other applications, such as neural and abdominal imaging.

In conclusion, we found that while agreement among the majority of ADC mapping implementations was good, the biases in *in vivo* ADC measures both between different offline implementations and between vendor-generated and offline maps are significant. Furthermore, these differences may, in some cases, be large enough to adversely affect the analysis of multisite diffusion data. For any given longitudinal (e.g., treatment response) or cross-sectional study, we would recommend that all analyses be performed on a common platform and that the output parametric map metadata reflect both the DWI data origin and the details of the applied calculation algorithm.
